# Retailer Adherence to Family Smoking Prevention and Tobacco Control Act, North Carolina, 2011

**DOI:** 10.5888/pcd10.120184

**Published:** 2013-04-04

**Authors:** Shyanika W. Rose, Allison E. Myers, Heather D’Angelo, Kurt M. Ribisl

**Affiliations:** Author Affiliations: Allison E. Myers, Heather D’Angelo, Kurt M. Ribisl, University of North Carolina, Chapel Hill, North Carolina.

## Abstract

**Introduction:**

The Family Smoking Prevention and Tobacco Control Act regulates the sales and marketing of tobacco products in the United States; poor adherence by tobacco retailers may reduce the effectiveness of the Act’s provisions. The objectives of this study were 1) to assess whether and to which provisions retailers were adherent and 2) to examine differences in adherence by county, retailer neighborhood, and retailer characteristics.

**Methods:**

We conducted multivariate analysis of tobacco retailers’ adherence to 12 point-of-sale provisions of the Tobacco Control Act in 3 North Carolina counties. We conducted observational audits of 324 retailers during 3 months in 2011 to assess adherence. We used logistic regression to assess associations between adherence to provisions and characteristics of each county, retailer neighborhood, and retailer.

**Results:**

We found 15.7% of retailers did not adhere to at least 1 provision; 84.3% adhered to all provisions. The provisions most frequently violated were the ban on sales of cigarettes with modified-risk labels (eg, “light” cigarettes) (43 [13.3%] retailers nonadherent) and the ban on self-service for cigarettes and smokeless tobacco (6 [1.9%] retailers nonadherent). We found significant differences in rates of nonadherence by county and type of retailer. Pharmacies and drug stores were more than 3 times as likely as grocery stores to be nonadherent.

**Conclusion:**

Most tobacco retailers have implemented regulatory changes without enforcement by the US Food and Drug Administration. Monitoring rates of adherence by store type and locale (eg, county) may help retailers comply with point-of-sale provisions.

## Introduction

In 2009, the Family Smoking Prevention and Tobacco Control Act (Tobacco Control Act) gave the US Food and Drug Administration (FDA) the power to regulate tobacco products, including the sales and marketing of cigarettes, smokeless tobacco products, and roll-your-own tobacco (1). The Tobacco Control Act contained 12 new provisions that changed the point-of-sale environment by restricting the sale of certain tobacco products, advertisements, and promotions and requiring specific product placement and labeling. The Tobacco Control Act focused on tobacco retailers in part because of concerns about point-of-sale advertising, marketing, and promotion of tobacco products to youth and in racial/ethnic minority communities (2). 

Tobacco retailers may contribute to disparities in tobacco use by increasing the availability of tobacco products and the number of environmental cues to smoke (3). The density of tobacco retailers (4–6) and level of tobacco advertising and promotion may be greater in racial/ethnic minority and low-income neighborhoods (7–12), and cigarette prices may be lower in racial/ethnic minority communities (13). Retailers may also contribute to disparities by differing in regulatory compliance according to neighborhood characteristics. Several studies have documented lower rates of compliance with provisions that address the access of minors to tobacco products in racial/ethnic minority and socioeconomically disadvantaged neighborhoods (14,15).

These 12 new provisions include bans on certain types of tobacco products (eg, flavored cigarettes), restrictions on advertising and labeling (eg, banning such “modified-risk” labels as “light,” “low tar” and “mild”), changes in sales practices (eg, requiring face-to-face, clerk-assisted sales of cigarettes and smokeless products instead of allowing self-service), and restrictions on promotions (eg, providing a gift with purchase).

Only 2 studies have examined the adherence of retailers to the new Tobacco Control Act point-of-sale provisions (16,17) or determined whether adherence varies by retailer neighborhood characteristics (16). One study examined 4 provisions and found no difference in adherence between retailers in high- and low-income neighborhoods in 1 county in Ohio (16). The other, which focused on sales and marketing of smokeless tobacco in 3 rural Appalachian Ohio counties, also examined 4 provisions and found significant decreases in provision of self-service cigarettes and smokeless tobacco (17). To our knowledge, no studies have reported on adherence to a comprehensive range of provisions. The objectives of this study were to 1) assess whether and to which provisions retailers were adherent, and 2) examine differences in adherence by county, retailer neighborhood, and retailer characteristics.

## Methods

We used multivariate logistic regression to analyze data from observational audits of a random sample of 324 tobacco retailers in 3 North Carolina counties (Buncombe, Durham, and New Hanover). We conducted the audits in September, October, and November 2011, before the introduction of advertising and labeling inspections by the FDA. The University of North Carolina at Chapel Hill (UNC–CH) Public Health–Nursing institutional review board determined that the study did not constitute human subjects research and thus did not require approval.

### Study setting and sample

Buncombe County is in Appalachia, Durham County is in the central part of the state in a racially and ethnically diverse urban area in which several universities are located, and New Hanover County is in an urban area on the Atlantic coast (Figure). We selected these counties to represent different geographic regions of North Carolina and to expand the demographic diversity of the data used to study retailer adherence. These 3 counties also represent a subset of North Carolina counties that have higher-than-average cancer rates and have been identified as priority counties by the University Cancer Research Fund.

North Carolina is one of 12 states that do not license tobacco retailers (18), and thus the state does not have a comprehensive list of tobacco retailers. We identified all tobacco retailers in the 3 counties by consulting and verifying data from ReferenceUSA (19) and adding new retailers as we worked in the field. We used 10 North American Industry Classification System (NAICS) codes (20) to identify potential tobacco retailers in the following categories: supermarkets or grocery stores; gas and convenience stores; convenience stores; pharmacies and drug stores; tobacco stores; warehouse clubs and supercenters; news dealers and newsstands; beer, wine, and liquor stores; discount department stores; and other gas stations. We used the same NAICS store type codes used by ReferenceUSA and verified these codes in the field; we assigned codes to additional retailers identified according to NAICS definitions. Using a handheld Garmin GPSMap 60Cx (Garmin International, Inc, Olathe, Kansas), we ascertained a global positioning system (GPS) fix for each retailer near its front door. We identified 671 tobacco retailers in 3 counties and selected 347 for audit through stratified random sampling proportionate to the number of retailers in each county. During the audits, we excluded 14 retailers for the following reasons: 3 were out of business, 7 did not sell tobacco to consumers, and 4 could not be located. Of the 333 remaining eligible retailers (ie, stores verified as in business and selling tobacco to consumers), we were able to ascertain geographic and demographic characteristics, but 9 were not audited: 5 refused to be audited, 2 audits were not completed because of safety considerations, and 2 retailers were not visited because of an auditor error. Thus, we obtained complete audit data for 324 retailers, a completion rate of 97% of eligible retailers and 93% of retailers in the sample.

**Figure:**
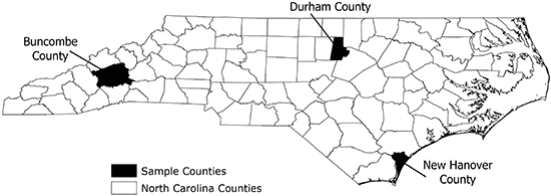
Three counties in North Carolina included in a study on retailer adherence to provisions of The Family Smoking Prevention and Tobacco Control Act.

### Measures

We developed an observational audit protocol that included measures of exterior and interior advertising and marketing similar to measures assessed in other studies (21–23); we also developed new measures. We focused on dichotomous measures, because these measures are more reliable in assessments by independent raters than subjective measures or measures of quantity or proximity (24).

We assessed adherence to the 12 point-of-sale provisions of the Tobacco Control Act that had been implemented at the time of the study. We categorized a retailer as adherent if the retailer met all of the following requirements (each corresponding to one of 12 provisions): 1) no sales of flavored cigarettes (excluding menthol); 2) no sales of cigarettes with such labels as “light,” “low tar,” “mild,” “ultra light,” and others (ie, modified-risk products); 3) no self-service for cigarettes or smokeless tobacco; 4) no tobacco vending machines; 5) no sales of loose cigarettes; 6) no sales of smokeless tobacco in less than a full package; 7) no audio or 8) video advertisements that have sound effects, music, or color; 9) no gifts with purchase; 10) no availability of gift catalogs; 11) no sales of branded nontobacco products; and 12) no promotion of tobacco brand–name event sponsorship. We classified retailers that did not adhere to 1 or more provisions as nonadherent. When a retailer sold modified-risk–labeled cigarettes, auditors recorded the brand name of all such cigarettes.

We assessed proximity of retailers to schools by using the retailer’s GPS position and county records of public K–12 school parcels. We created a dichotomous measure to code retailers as more than 1,000 feet from or within 1,000 feet of a school parcel boundary.

We defined neighborhoods as census tracts (25). Using a geographic information system (ArcGIS [Esri, Redlands, California]), we linked the retailer GPS position (latitude and longitude) to the following neighborhood characteristics: the percentage of black residents and the percentage of Hispanic residents, derived from 2010 US census data (26); the percentage of families living below federal poverty guidelines, based on the 2006–2010 American Community Survey (27) 5-year estimates; the percentage of residents who have a bachelor’s degree or more (27); and percentage of the population residing in rural areas, defined as fewer than 2,500 people in a given area (26). We obtained data on county smoking rates from the North Carolina State Center for Health Statistics (28).

### Procedures

For the audit phase, we trained 2 or 3 master’s degree–level data collectors per county through a day-long didactic and hands-on session conducted in retailer locations (a grocery store, a pharmacy, and a tobacco store) in a county not selected for the study. As part of a larger study on food, tobacco, and physical activity environments, 2 data collectors visited each retailer, but only one conducted the tobacco audit. We conducted all retailer audits electronically using data collection forms programmed in Pendragon (Pendragon Software Corporation, Buffalo Grove, Illinois) on an iPod touch (Apple Inc, Cupertino, California). We regularly reviewed data, which were uploaded to a secure central database.

### Analyses

To assess whether retailer and neighborhood characteristics were associated with retailer adherence, we used χ^2^ tests and logistic regression. The regression analyses used robust standard errors, applied with Proc Surveylogistic (SAS Institute Inc, Cary, North Carolina) to account for the stratified sampling design, and controlled for retailer characteristics, retailer neighborhood characteristics, and county characteristics. We categorized warehouse clubs and supercenters as supermarkets and grocery stores because they were full-service grocery stores (even if they also sold other products). We combined gas and convenience stores and other gas stations into 1 category. We found no news dealers or newsstands. Although we assessed neighborhood characteristics by census tract, almost 30% of census tracts had only 1 retailer; thus, our data were not hierarchical, and our analysis did not need to account for clustering of retailers by census tract. In multivariate analyses, we used the retailer type (supermarkets and grocery stores) and the county (Durham) that had the lowest rates of nonadherence as reference categories. We conducted all analyses using SAS version 9.2. 

## Results

Of the 333 retailers in the sample, 51.1% were gas and convenience stores, and 13.5% were convenience stores (Table 1). We found no significant differences in the distribution of retailer type by county.

**Table 1:** Characteristics of Tobacco Retailers (N = 333), Retailer Neighborhoods, and Counties, 3 North Carolina Counties and State, 2011

Characteristic	Comparison Group			
	**Buncombe County (n = 116)**	**Durham County (n = 115)**	**New Hanover County (n = 102)**	**Counties Combined (n = 333)**
**Product type sold, no. (%) of retailers^a^**				
Cigarettes	116 (100)	111 (100)	97 (100)	324 (100)
Smokeless tobacco	102 (87.9)	83 (74.8)	74 (76.3)	259 (79.9)
**Retailer type, no. (%) of retailers**				
Supermarket or grocery store	20 (17.2)	21 (18.3)	18 (17.7)	59 (17.7)
Gas and convenience	66 (56.9)	60 (52.2)	44 (43.1)	170 (51.1)
Convenience	9 (7.8)	19 (16.5)	17 (16.7)	45 (13.5)
Pharmacy or drug store	13 (11.2)	12 (10.4)	13 (12.8)	38 (11.4)
Tobacco store	4 (3.5)	2 (1.7)	7 (6.9)	13 (3.9)
Other^b^	4 (3.5)	1 (0.9)	3 (2.9)	8 (2.4)
**Retailer proximity to school, no. (%) of retailers**				
>1,000 feet from school	14 (12.1)	25 (21.7)	14 (13.7)	53 (15.9)
≤1,000 feet from school	102 (87.9)	90 (78.3)	88 (86.3)	280 (84.1)
**Retailer neighborhood characteristic, mean (SD), %**				
Black residents	6.4 (8.7)	41.5 (21.4)	19.1 (19.3)	22.6 (23.0)
Hispanic residents	6.5 (5.2)	16.1 (10.0)	6.1 (5.0)	9.7 (8.5)
Families living below poverty guidelines	10.6 (8.0)	15.7 (15.0)	12.1 (13.3)	12.8 (12.7)
Residents who have bachelor’s degree or more	29.9 (12.9)	36.0 (20.7)	32.0 (14.5)	32.7 (16.7)

**Characteristic**	**Buncombe County**	**Durham County**	**New Hanover County**	**North Carolina**

Smoking rate, %^c^	14.6	14.7	15.1	19.8
Median household income, $^d^	44,190	49,894	48,553	45,570
Black residents, %^d^	6.4	38.0	14.8	21.5
Hispanic residents, %^d^	6.0	13.5	5.3	8.4
Families living below poverty guidelines, %^e^	14.7	16.1	15.4	15.5
Residents who have bachelor’s degree or more, %^d^	31.2	44.1	31.6	26.1
Rural population, %^d^	24.1	5.6	2.2	33.9

Abbreviations: SD, standard deviation.

a Data were missing for 9 retailers (4 retailers in Durham County and 5 retailers in New Hanover County), so values are based on 324 retailers.

b Other retailer types included beer, wine, and liquor stores and discount department stores.

c North Carolina State Center for Health Statistics (28).

d US Census Bureau (26).

e 2006–2010 American Community Survey (27).

The 3 study counties were less rural than the state as a whole, and Durham County was more racially and ethnically diverse than the other 2 counties (Table 1). Retailer neighborhood characteristics varied by county. Compared with retailers in the other 2 counties, Durham County retailers were in neighborhoods that had a significantly higher percentage of black (41.5%) and Hispanic (16.1%) residents, and a higher percentage (15.7%) of residents living below federal poverty guidelines. Durham County retailers were also in neighborhoods that had a significantly higher percentage (36.0%) of residents who had a bachelor’s degree or more than had Buncombe County retailers (29.9%) but not New Hanover retailers (32.0%).

Among the 3 counties, 15.7% of retailers violated at least 1 point-of-sale provision (Table 2). Of the 51 nonadherent retailers, 4 violated 2 provisions, and 47 violated 1 provision. The provisions most frequently violated were the ban on sales of cigarettes with modified-risk labels (43 [13.3%] retailers nonadherent) and the ban on self-service for cigarettes and smokeless tobacco (6 [1.9%] retailers nonadherent). Of the 43 retailers selling cigarettes with modified-risk labels, 22 sold only 1 brand, and the rest sold 2 or more. We identified 26 cigarette brands with modified-risk labeling, of which Phillip Morris brands were predominant. The most common modified-risk brands were Basic (sold by 33 retailers), Marlboro (sold by 11 retailers), Virginia Slims (sold by 10 retailers), and Parliament (sold by 5 retailers). The remaining 22 modified-risk brands were carried by only 1 or 2 retailers. We found sales of flavored cigarettes, branded nontobacco products, smokeless tobacco sold in less than a full pack, promotion of brand-name event sponsorship, or gifts with purchase in only 1 or 2 retailers. We found no violations of the bans on tobacco vending machines, sales of loose cigarettes, availability of gift catalogs, or audio and video advertisements. Counties differed by violation rate: 34.5% of Buncombe County retailers were nonadherent, whereas only 2.7% of Durham County retailers and 8.3% of New Hanover County retailers were nonadherent.

**Table 2:** Characteristics of Retailers (N = 324) That Did Not Adhere to At Least 1 Provision of Tobacco Control Act, by County, North Carolina, 2011

Characteristic	Buncombe County (n = 116)	Durham County (n = 111)	New Hanover County (n = 97)	Combined (n = 324)
**Did not adhere to at least 1 provision, n (%)**	40 (34.5)	3 (2.7)	8 (8.3)	51 (15.7)
**Retailer type, no. of retailers (% of retailer type^a^)**				
Supermarket or grocery store	4 (20.0)	1 (4.8)	1 (6.3)	6 (10.5)
Gas and convenience	22 (33.3)	2 (3.5)	2 (4.9)	26 (15.9)
Convenience	2 (22.2)	0	3 (17.7)	5 (11.4)
Pharmacy or drug store	9 (69.2)	0	0	9 (23.7)
Tobacco store	2 (50.0)	0	2 (28.6)	4 (30.8)
Other^b^	1 (25.0)	0	0	1 (12.5)
**Retailer proximity to school, no. of retailers (% in category of school proximity^c^)**				
>1,000 feet from school	5 (35.7)	1 (4.2)	0	6 (11.7)
≤1,000 feet from school	35 (34.3)	2 (2.3)	8 (9.5)	45 (16.5)
**Provision violated, no. (%) of retailers**				
No sales of cigarettes claiming modified risk (labels such as “light” or “low tar”)	38 (32.8)	2 (1.8)	3 (3.1)	43 (13.3)
No self-service for cigarettes or smokeless tobacco	2 (1.7)	1 (0.9)	3 (3.1)	6 (1.9)
No sales of flavored cigarettes	0	0	2 (2.1)	2 (0.6)
No promotion of tobacco brand–name event sponsorship	0	0	1 (1.0)	1 (0.3)
No sales of branded nontobacco products	1 (0.9)	0	0	1 (0.3)
No sales of smokeless tobacco in less than full package	1 (0.9)	0	0	1 (0.3)
No gifts with purchase	1 (0.9)	0	0	1 (0.3)
No tobacco vending machines	0	0	0	0
No sales of loose cigarettes	0	0	0	0
No availability of gift catalogs	0	0	0	0
No audio advertisements that have sound effects or music	0	0	0	0
No video advertisements that have sound effects, music, or color	0	0	0	0

a Percentages are based on the number of retailers per county for each category of retailer type (Table 1).

b Other retailer types included beer, wine, and liquor stores and discount department stores.

c Percentages are based on the number of retailers per county for each category of school proximity (Table 1)

No retailer type was significantly more likely to be nonadherent than our reference group, supermarkets or grocery stores (Model 1), even after factoring in retailer neighborhood characteristics (Model 2) (Table 3). Certain retailer neighborhood characteristics were significantly associated with the likelihood of adherence. Retailers in neighborhoods with a greater percentage of black residents were 7% less likely to be nonadherent, whereas retailers in neighborhoods with a greater percentage of families living below federal poverty guidelines were 8% more likely to be nonadherent. Retailer proximity to schools was not associated with likelihood of adherence. 

**Table 3:** Odds Ratios and 95% Confidence Intervals for Any Violation of Point-of-Sale Provisions of Tobacco Control Act, North Carolina, 2011^a^

Effect	Model 1: Retailer Characteristics	Model 2: Retailer and Retailer Neighborhood Characteristics	Model 3: Retailer, Retailer Neighborhood, and County Characteristics
**Retailer type**			
Supermarket or grocery store	1.00 [Reference]		
Gas and convenience	1.60 (0.62–4.16)	1.73 (0.65–4.57)	1.60 (0.57–4.47)
Convenience	1.09 (0.31–3.88)	1.35 (0.32–5.76)	1.65 (0.32–8.66)
Pharmacy or drug store	2.64 (0.85–8.21)	3.37 (0.97–11.69)	3.44 (1.07–11.06)
Tobacco store	3.78 (0.87–16.40)	3.92 (0.91–16.92)	4.74 (0.79–28.56)
Other^a^	1.21 (0.13–11.95)	0.78 (0.06–10.26)	0.73 (0.05–10.97)
**Retailer neighborhood characteristics**			
Percentage of black residents	—	0.93 (0.89–0.96)	0.96 (0.92–1.00)
Percentage of families living below poverty guidelines	—	1.08 (1.03–1.14)	1.06 (1.00–1.12)
Percentage of residents who have bachelor’s degree or more	—	0.99 (0.96- 1.01)	1.00 (0.98–1.04)
Percentage of Hispanic residents	—	0.97 (0.92–1.04)	0.99 (0.91–1.07)
Retailer located >1,000 feet from school	1.00 [Reference]		
Retailer located ≤1,000 feet from school	—	0.95 (0.32–2.83)	0.92 (0.30–2.77)
**County**			
Durham	1.00 [Reference]		
New Hanover	—	—	1.5 (0.33–6.66)
Buncombe	—	—	7.42 (1.74–31.71)

a Other retailer types included beer, wine, and liquor stores and discount department stores.

Retailers in Buncombe County were 7.4 times as likely to be nonadherent as retailers in Durham County (Model 3). We found no difference in adherence between Durham County and New Hanover County retailers. After controlling for county characteristics, we found that retailer neighborhood demographics were no longer significantly associated with adherence but that pharmacies and drug stores were 3.4 times as likely as supermarkets or grocery stores to be nonadherent.

## Discussion

This study is one of only 3 to examine adherence to Tobacco Control Act point-of-sale provisions 1 or 2 years after the provisions were enacted, and the first to report on all 12 point-of-sale provisions that were in effect in 2011. Our study found that 15.7% of tobacco retailers were nonadherent to point-of-sale provisions, whereas 2 previous studies found nonadherence rates below 10% (16,17). Our higher rate may be explained by the greater number of point-of-sale provisions included in our study. From January to August 2012, the FDA conducted compliance checks in 36 states (including North Carolina) and issued warning letters or civil penalty letters in 5% of the checks (29). The 2 previous studies identified violation rates of 3% to 8% for bans on self-service of cigarettes and smokeless tobacco products (16,17), whereas our study found violations of this ban in only 1.9% of retailers. We found that the provision most frequently violated was the ban on sales of modified-risk cigarettes, a provision not yet enforced by the FDA. These types of cigarettes were banned under the Tobacco Control Act starting in June 2010. Retailers are allowed to sell existing stock, but they were asked to voluntarily remove these products from the market (30). However, in some areas, these types of cigarette packs were found to be available more than 15 months after the ban. It seems reasonable that the FDA could now consider enforcing this provision.

After controlling for county differences, we did not observe disparities in adherence by retailer neighborhood characteristics such as percentage of families living below federal poverty guidelines, resident educational levels, percentage of black or Hispanic residents, or retailer proximity to a school. Other studies have found differential adherence by neighborhood characteristics to restrictions on access to minors (14) and on tobacco retailer advertising and promotion (3,31).

After controlling for an array of neighborhood and county factors, however, we found that pharmacies and drug stores were significantly more likely to be nonadherent than any other retailer type. Tobacco products account for only 1.8% of total pharmacy sales (32), and 3.2% of all tobacco sales occur at pharmacies (K.M.R., unpublished data, 2012). Thus, pharmacies may be less aware of or affected by new point-of-sale sales provisions than other retailer types. Several researchers have called for a ban on sales of tobacco products by pharmacies because they are typically associated with health promotion (32,33). Our finding that pharmacies are more likely to be nonadherent to point-of-sale provisions may provide another argument in favor of a ban on tobacco sales in pharmacies.

Our study has several strengths. It demonstrated relatively strong adherence to Tobacco Control Act provisions before the FDA implemented formal compliance checks. Thus, this study provides baseline compliance data. We also examined more provisions than were examined in previous studies (16,17). Our study also has several limitations. The counties in our sample were geographically and demographically diverse, but our findings may not be generalizable beyond North Carolina or the region. We also may have lacked the power to detect neighborhood-level differences when we controlled for county characteristics because we included only 3 counties. A broader national study, using similar methods to those in this study, is planned to address these limitations. Different teams of researchers collected data in the different counties, so we cannot wholly rule out auditor effects in the differences detected between counties. Additionally, we were unable to conduct tests of inter-rater reliability because of resource constraints. However, data collectors were centrally trained and monitored, and analysis of other measures (data for which were not presented in this study) did not significantly differ among data collectors. Future attention should be paid to methods to standardize data collection measures and assess their reliability. Finally, because the study was cross-sectional, we were unable to demonstrate changes in adherence that occurred before implementation of the Tobacco Control Act.

Our findings indicate that most tobacco retailers have implemented regulatory changes without substantial enforcement by the FDA. However, some patterns remain: high rates of nonadherence to the ban on sales of cigarettes labeled as modified risk, higher likelihood of nonadherence among pharmacies and drug stores than among other retailer types, and the potential for differential adherence by county. Encouragingly, we found little evidence of disparities in adherence to point-of-sale sales and marketing provisions of the Tobacco Control Act in racial/ethnic minority or low-income neighborhoods. As formal mechanisms for compliance checking go into effect, we anticipate that adherence will improve. We recommend monitoring for differential adherence across point-of-sale provisions, retailer types, and locales as a strategy to strengthen the ability and intention of retailers to adhere to Tobacco Control Act regulations.
